# *Helicobacter pylori* eradication: a narrative review

**DOI:** 10.3389/fgstr.2026.1823571

**Published:** 2026-07-01

**Authors:** Tarig Fadelelmoula, Hamdi Al Mutori, Khalid Mohammed, Mazin Saleh, Ali Al Reesi

**Affiliations:** 1 Department of Medicine, College of Medicine and Health Sciences, National University of Science and Technology, Sohar, Oman; 2 Department of Internal Medicine, Sohar Hospital, Sohar, Oman

**Keywords:** antibiotic resistance, eradication, gastric cancer, global health, *Helicobacter pylori*, probiotics

## Abstract

**Background:**

*Helicobacter pylori* (*H. pylori*) is one of the most common chronic infections worldwide and a primary cause of peptic ulcer disease and gastric cancer. Increasing antibiotic resistance has made eradication more difficult, emphasizing the need for new strategies. This narrative review summarizes the current evidence on *H. pylori* eradication, focusing on epidemiology, resistance, treatment regimens, adjunctive therapies, cancer prevention, and extra-gastric outcomes.

**Evidence and methods:**

We conducted a narrative literature search to identify current evidence on strategies for eradicating *H. pylori*. Both first-line and salvage regimens were considered, as well as adjunctive and novel approaches. The evidence was organized thematically.

**Results:**

Over half of the world’s population is infected with *H. pylori*, with the highest prevalence observed in Africa, South America, and Asia. Clarithromycin resistance exceeds 15%-20% in most regions, which diminishes the effectiveness of standard triple therapy. Current recommendations for first-line treatments in areas with high resistance include bismuth quadruple and concomitant regimens, which achieve eradication rates above 85%. Vonoprazan-based and high-dose dual therapies also show promise as alternative options. For second-line therapy, levofloxacin- and bismuth-based regimens remain effective, while rifabutin and susceptibility-guided approaches offer salvage options. Additionally, probiotics and N-acetylcysteine have been found to improve eradication rates and treatment tolerability. Successful eradication reduces the risk of gastric cancer, particularly in East Asia, and provides benefits for extra-gastric conditions such as iron deficiency anemia, idiopathic thrombocytopenic purpura, and pregnancy-related complications.

**Conclusion:**

Eradicating *H. pylori* is crucial for preventing gastric cancer and enhancing gastrointestinal and systemic health. Achieving success necessitates adapting treatment to resistance patterns, employing adjunctive strategies, using molecular antimicrobial sensitivity testing, and implementing eradication programs in areas with high prevalence.

## 1 Introduction

*Helicobacter pylori* (*H. pylori*) is a gram-negative bacterium that colonizes the gastric mucosa and is among the most common chronic bacterial infections worldwide (1). The global prevalence is estimated to exceed 4 billion people, with the highest rates reported in Africa, South America, and Asia, and lower but declining rates in Europe and North America (2, 3). Transmission usually occurs during childhood and is strongly influenced by socioeconomic conditions, sanitation, and household crowding (4, 5).

Since its identification, *H. pylori* has been firmly established as the primary cause of chronic gastritis, peptic ulcer disease, and gastric malignancies, including adenocarcinoma and mucosa-associated lymphoid tissue (MALT) lymphoma (6, 7). Gastric cancer is among the leading causes of cancer-related mortality worldwide, and eradication of *H. pylori* has been recognized as a crucial preventive strategy, particularly in high-prevalence populations (8, 9). Beyond gastric disease, infection has also been linked to iron deficiency anemia and idiopathic thrombocytopenic purpura, underscoring its systemic impact (10, 11).

In the 1990s, early eradication methods, notably the combination of clarithromycin-based triple therapy with a proton pump inhibitor (PPI) plus either amoxicillin or metronidazole, showed high success rates (12). However, increasing antibiotic resistance has diminished their effectiveness, with clarithromycin resistance surpassing 15-20% in many regions and dual resistance to clarithromycin and metronidazole becoming more common (13–17). Consequently, international guidelines now advise against using standard triple therapy in areas with high resistance levels (18).

Alternative regimens, including bismuth quadruple therapy, concomitant therapy, and sequential therapy have proven more effective in eradication under resistant conditions (19, 20). Complementary strategies, such as probiotics to reduce side effects and boost adherence, and N-acetylcysteine to break down bacterial biofilms, have also shown potential to improve eradication rates (22–24).

The continued high global prevalence, the vital role of *H. pylori* in gastric carcinogenesis (8, 9), and the increasing problem of antibiotic resistance highlight the need to optimize eradication strategies (4). Successful eradication is essential not only for individual patient outcomes but also for lowering the worldwide burden of gastric cancer (7–9).

Despite years of clinical experience and various consensus guidelines, managing *H. pylori* remains complex. This complexity arises from evolving antibiotic resistance, regional variation in eradication success, and limited availability of susceptibility-guided therapy in many areas. Therefore, an updated review is needed to analyze current evidence on resistance patterns, treatment options, and new diagnostic techniques, and to identify existing gaps in clinical practice and research.

## 2 Methodology

This review aims to provide a narrative synthesis of the current evidence concerning eradication strategies for *H. pylori*. Its goal is to offer a comprehensive overview of epidemiology, patterns of antibiotic resistance, therapeutic regimens, adjunctive interventions, and clinical outcomes of eradication, including both gastric and extragastric effects.

### 2.1 Sources of evidence

This narrative review was founded on an extensive literature search of PubMed and related bibliographic sources, concentrating on articles published in English.

### 2.2 Search approach

The search strategy involved combining key terms such as “*Helicobacter pylori*,” “eradication,” “antibiotic resistance,” “treatment regimens,” “susceptibility testing,” and “guidelines.” Priority was given to recent systematic reviews, meta-analyses, international consensus reports, and large observational studies, especially those published after 2020. Additional relevant articles were located through manual screening of reference lists.

### 2.3 Eligibility criteria

As this was a narrative review, formal inclusion and exclusion criteria, as well as quantitative synthesis, were not applied.

## 3 Results

### 3.1 Global epidemiology of *H. pylori*

Earlier estimates suggested that more than half of the global population was infected with *H. pylori*. Recent research, however, shows that the prevalence is gradually declining and is now below 50% worldwide (1). This reduction is not uniform, with high infection rates remaining in many low- and middle-income regions, particularly in parts of Africa, South America, and Asia, while Western Europe and North America have much lower rates (1–3). These regional disparities have important consequences for eradication strategies and the development of antibiotic resistance (4).

Longitudinal studies have revealed significant effects linked to birth cohorts. In Japan, infection rates dropped from over 70% among those born before 1950 to under 20% among those born after 1980, reflecting improved living conditions and sanitation (5). Similar trends are observed in Europe and North America, where prevalence declines continue with socioeconomic improvements (2, 6). Nonetheless, *H. pylori* persists in younger populations within developing countries, where infection remains common (4, 7). In regions with high prevalence, universal screening and eradication are considered strategies for cancer prevention at the population level, though their feasibility and cost-effectiveness vary by region (8, 9). **Table 1** outlines the global epidemiological trends and prevalence.

**Table 1:** Global prevalence and epidemiological trends of *H. pylori.*.

Region	Prevalence (%)	Key notes	Reference
Africa	>70	Highest global prevalence	(1, 2)
South America	>70	High prevalence	(1, 2)
Asia	>70	High prevalence, declining in some areas	(1, 5)
Europe	<40	Declining trends	(2, 6)
North America	<40	Low and declining	(2)

### 3.2 Antibiotic resistance and its clinical implications

Antibiotic resistance remains the primary challenge to successful eradication. Resistance to clarithromycin, metronidazole, and levofloxacin has risen sharply over the last 20 years and now prevails across all WHO regions (12–16). Most areas report clarithromycin resistance over 15%-20%, with the highest levels observed in the Asia-Pacific and Latin America (15, 16). European surveillance has demonstrated strong correlations between macrolide use and clarithromycin resistance, which diminishes the efficacy of triple therapy (14). In Iran, studies in children indicate metronidazole resistance rates over 60% (15). **Table 2** shows global and regional resistance rates of *H. pylori* to antibiotics. Resistance to clarithromycin and levofloxacin is strongly associated with eradication failure (13). Conversely, therapy guided by susceptibility testing improves eradication rates compared to empirical approaches, supporting local or personalized resistance testing whenever feasible (17). The Maastricht IV and V consensus reports advise against using clarithromycin-based triple therapy in areas where resistance exceeds 15%, favoring bismuth quadruple or concomitant therapies instead (18).

**Table 2:** Global and regional *H. pylori* antibiotic resistance rates.

Antibiotic	Global resistance (%)	Highest resistance regions	Notes	References
Clarithromycin	15 - 20	Asia-Pacific, Latin America	Key driver of therapy failure	(11, 12)
Metronidazole	40 - 60	Middle East, Iran (children)	Common dual resistance	(15)
Levofloxacin	20 - 30	Asia, Europe	Rising trend globally	(14, 16)

### 3.3 First-line and alternative eradication therapies

For nearly two decades, clarithromycin-based triple therapy (PPI, clarithromycin, and amoxicillin or metronidazole) was the standard first-line regimen (12, 19). Initial studies demonstrated eradication rates exceeding 80-90% (12, 20). However, increasing resistance has diminished its effectiveness, with cure rates often dropping below 70% in areas with high resistance (4, 5).

Sequential therapy, which involves a two-phase eradication regimen where a proton pump inhibitor and amoxicillin are administered initially, followed by a combination of a proton pump inhibitor with clarithromycin and a nitroimidazole in the second phase, has shown modest superiority over triple therapy in early trials, but its benefit is reduced in dual-resistant strains (21). Concomitant therapy (PPI, clarithromycin, amoxicillin, and metronidazole) consistently achieves higher eradication rates than triple therapy, with similar tolerability across regimens (19).

Bismuth quadruple therapy (PPI, bismuth, tetracycline, and metronidazole) has re-emerged as a highly effective first-line treatment, especially in regions where clarithromycin resistance exceeds 15-20% (17). Network meta-analyses confirm that both bismuth quadruple and concomitant regimens surpass triple therapy, with eradication rates consistently above 85% (19). High-dose dual therapy, which combines potent acid suppression with high-dose amoxicillin, and Vonoprazan-based triple therapy, also show promising results (27). Current consensus guidelines recommend bismuth quadruple or concomitant therapy as first-line options in areas of high resistance, reserving triple therapy only for regions with demonstrably low resistance (18). **Table 3** compares the outcomes of the primary eradication regimens.

**Table 3:** Comparative outcomes of first-line eradication regimens.

Regimen	Eradication rate (%)	Notes	References
Triple therapy	<70	Falling efficacy due to resistance	(12, 19)
Sequential therapy	70-80	Limited advantage in dual resistance	(22)
Concomitant therapy	>85	Superior to triple, good tolerability	(10)
Bismuth quadruple	>85	Recommended in high-resistance settings	(9, 18)
High-dose dual	80-85	Dependent on strong acid suppression	(13)
Vonoprazan triple	>90	Enhanced efficacy vs PPI regimens	(29)

### 3.4 Second line and rescue therapies

Failure to eradicate after initial therapy necessitates effective salvage regimens. Levofloxacin-based triple therapy (PPI, levofloxacin, and amoxicillin) achieves high eradication rates but is hindered by the increasing prevalence of fluoroquinolone resistance (13, 15, 16). Bismuth-containing regimens remain effective in salvage settings, even after exposure to clarithromycin or fluoroquinolones (17). Rifabutin-based therapy offers an additional option following multiple failures. However, concerns about toxicity and tuberculosis resistance limit its use (19). Quinolone-containing rescue regimens can achieve eradication rates above 70% when carefully selected (13). Tailored therapy guided by antimicrobial susceptibility or molecular testing consistently improves success rates and is recommended where available (18). In **Table 4** below, we present the eradication rates for the second line and rescue medications.

**Table 4:** Second-line and rescue regimen for *H. pylori* eradication.

Regimen	Eradication rate (%)	Notes	References
Levofloxacin triple	70-80	Resistance limits efficacy	(15–17)
Bismuth-containing	>80	Effective after failure of other regimens	(18)
Rifabutin-based	70-80	Toxicity and TB resistance risk	(19)
Tailored therapy	>85	Guided by susceptibility testing	(21)

### 3.5 Adjunctive and novel strategies

Adjunctive and novel therapies aim to optimize eradication outcomes. Probiotics (Lactobacillus, Bifidobacterium) may enhance eradication rates and reduce treatment-related gastrointestinal side effects, such as diarrhea and nausea (22, 23). Mechanisms include competitive inhibition of *H. pylori*, modulation of gut microbiota, and improvement of mucosal immunity (24).

N-acetylcysteine (NAC), a biofilm disruptor, enhances eradication success when added to standard regimens (25, 26). Host genetic factors influence outcomes. CYP2C19 polymorphisms impact PPI metabolism, while IL-1B variants modulate gastric acid secretion, both contributing to variability in treatment results (27, 28). Vonoprazan, a potassium-competitive acid blocker, has demonstrated superior acid suppression compared to PPIs, improving antibiotic efficacy and achieving higher eradication rates in randomized trials (27).

### 3.6 Cancer prevention

Gastric cancer develops through a multistep process from chronic gastritis to carcinoma (29). *H. pylori* eradication breaks this sequence and lowers cancer risk. Randomized controlled trials and meta-analyses confirm considerable reductions in gastric cancer following eradication, especially in East Asian populations (8, 9, 30–32). The greatest benefits occur when eradication happens before precancerous changes, but even treatment later can slow disease progression (29). Population-level screening and eradication efforts in Japan and Korea show that these strategies are feasible and can be cost-effective in regions with high prevalence (2, 33).

### 3.7 Extra-gastric benefits of eradication

Eradication offers benefits beyond the stomach. It improves hematological parameters in cases of unexplained iron-deficiency anemia, especially in children and women (10, 34). Platelet recovery is observed in some patients with idiopathic thrombocytopenic purpura (11, 35).

In functional dyspepsia, eradication leads to modest symptom improvement, although benefits vary (36, 37). Eradication has been associated with improvements in systemic sclerosis and may reduce pregnancy complications linked to *H. pylori*, including micronutrient deficiencies and gestational diabetes mellitus (38–40). Connections with colorectal cancer and irritable bowel syndrome remain weaker and inconsistent (41, 42). **Table 5** summarizes the extra-gastric benefits of *H. pylori* eradication.

**Table 5:** Evidence of extra-gastric benefits of *H. pylori* eradication.

Condition	Effect of eradication	Notes	References
Iron deficiency anemia	Improves hemoglobin and iron levels	Particularly in children and women	(34)
Idiopathic Thrombocytopenic Purpura	Platelet recovery in subsets	Response variable	(35)
Functional dyspepsia	Modest symptom improvement	Not universal	(37)
Systemic sclerosis	Improved clinical outcomes	Association reported	(38)
Pregnancy-related	Reduces micronutrient deficiency, GDM risk	Observed in systematic reviews	(39, 40)
Colorectal cancer	Association suggested	Evidence inconsistent	(41)
Irritable Bowel Syndrome	Weak/uncertain effect	Evidence limited	(42)

## 4 Discussion

The eradication of *H. pylori* remains a fundamental element of gastrointestinal and cancer prevention strategies; however, the increasing prevalence of antibiotic resistance has fundamentally changed the therapeutic landscape. Early clarithromycin-based triple therapy achieved eradication rates of over 80% in the 1990s (12, 20). But today, global clarithromycin resistance exceeds 15-20% in most regions, making standard triple therapy ineffective in many populations (11, 14–16). This has prompted significant updates in international guidelines, which now recommend abandoning clarithromycin-based regimens in high-resistance areas and instead prioritizing bismuth quadruple or concomitant therapy (18).

The most significant obstacle to effective eradication is the global rise in resistance to clarithromycin, metronidazole, and levofloxacin (10–13, 16). Surveillance studies clearly demonstrate connections between regional antibiotic use and resistance rates, with macrolide consumption driving clarithromycin resistance in Europe (14). Pediatric cohorts in Iran highlight the serious challenge posed by metronidazole resistance, which exceeds 60% (15). Since eradication failure is closely linked to resistance, susceptibility-guided therapy has become an effective approach, improving cure rates compared with empirical treatment (17). However, access to molecular or culture-based resistance testing remains limited in many settings.

First-line therapy has evolved considerably in response to resistance pressures. Concomitant therapy and bismuth quadruple regimens consistently achieve eradication rates above 85% (9, 10, 18). Vonoprazan-based regimens further improve efficacy by offering superior acid suppression compared with proton pump inhibitors (27, 29). High-dose dual therapy with amoxicillin and potent acid inhibition has also shown promise in specific populations (13). These regimens remain the most viable options for maintaining eradication success globally.

Second-line and rescue strategies remain vital for patients who have had previous treatment failures. Levofloxacin-based regimens offer moderate effectiveness (15–17). Although increasing resistance to fluoroquinolones threatens their long-term effectiveness. Rifabutin-based therapy provides an additional option but carries risks of hematemesis toxicity and concerns about tuberculosis resistance (19). Tailored therapy guided by antimicrobial susceptibility or molecular testing remains the most effective salvage approach where available (21).

Probiotics increase eradication rates and reduce gastrointestinal side effects (22–24). NAC disrupts bacterial biofilms, enhancing antibiotic penetration and efficacy (25, 26). Host genetic factors also play a role, with CYP2C19 polymorphisms influencing the metabolism of proton pump inhibitors and IL-1B variants affecting gastric acid secretion (26). Personalized approaches that integrate host genetics and antimicrobial susceptibility are likely to shape future treatment paradigms.

The preventive effect of eradication on gastric cancer is now well established. Randomized controlled trials and meta-analyses confirm that *H. pylori* eradication significantly reduces gastric cancer incidence, particularly in high-risk Asian populations (28–33). Importantly, eradication is most beneficial when performed before the development of precancerous lesions, although benefits persist even at later stages (32). Population-level programs in Japan and Korea demonstrate the feasibility of mass screening and eradication as strategies to prevent cancer.

Beyond gastric disease, eradication offers benefits for several extra-gastric conditions. Evidence supports improvements in iron deficiency anemia and idiopathic thrombocytopenic purpura (34, 35). Outcomes in functional dyspepsia remain inconsistent, although modest improvements have been reported (36, 37). Eradication has also been linked to improvements in systemic sclerosis (38). A decreased risk of micronutrient deficiency and gestational diabetes during pregnancy has also been reported (39, 40). There is a potential association with colorectal cancer and irritable bowel syndrome; however, these connections are weak and require further research (41, 42).

## 5 Conclusion

*H. pylori* eradication is one of the most effective methods to prevent serious gastric diseases and improve patient outcomes. However, the rapid increase in antibiotic resistance has made treatment more difficult, emphasizing the need to move away from a “one-size-fits-all” approach. It is crucial to select therapies that account for local resistance patterns, use newer, more effective regimens, and incorporate adjunctive strategies to help restore high cure rates. Looking ahead, enhanced surveillance, greater access to customized therapies, and well-planned public health programs in high-prevalence areas will be essential in reducing the global burden of *H. pylori* and its complications. Increased utilization of Molecular Antimicrobial Sensitivity Testing should be a priority to inform future treatment options.

### 5.1 Review limitation

As a narrative synthesis, this review does not provide quantitative comparisons or a formal risk-of-bias assessment. In addition, regional data on antibiotic resistance remain incomplete, limiting the ability to draw country-specific conclusions. Nevertheless, compared with recently published reviews, this manuscript emphasizes the clinical implications of evolving resistance patterns and emerging diagnostic technologies, highlighting practical considerations for everyday practice.
